# Comparative analysis in selecting best irrigation method to maximize tomato yield from various irrigation approaches in water scarce regions

**DOI:** 10.1016/j.heliyon.2024.e28746

**Published:** 2024-04-02

**Authors:** Solomon Mathewos Boltana, Tigistu Yisihak Ukumo, Tarun Kumar Lohani, Nahom Bekele Mena, Muluneh Legesse Edamo, Matusal Arja Alaro, Bereket Dora Doliso

**Affiliations:** a Faculty of Water Resources and Irrigation Engineering, Arba Minch University, Ethiopia; b College of Engineering, Bule Hora University, Bule Hora, Ethiopia; c Faculty of Hydraulic and Water Resources Engineering, Arba Minch University, Ethiopia

**Keywords:** Evapotranspiration method, Irrigation scheduling, Soil moisture method, Tomato, Yield attributes

## Abstract

It is now essential for farmers to evaluate and compare different irrigation scheduling strategies in order to operate their irrigation systems in accordance with their resources and achievements. For tomato crops, a rigorous comparison was made between the yield of soil moisture and irrigation scheduling systems based on evapotranspiration. The experimental setup used a randomized complete block design (RCBD) with three replications, with water requirements of 100%, 75%, and 50%. Using a ReplogleBos-Clemmens (RBC) flume with an input rate of 1.62 l/s, water was applied to the furrows. The total applied amount in the Soil Moisture (SM) and Evapotranspiration (ET) based methods was 229.1 mm, and 280 mm, respectively. The collected data were assessed using ANOVA at a 5% significance level. For every technique, yield values, yield attributes, and agronomic traits were computed. The result indicates that there is no appreciable variation among the factors. The net yield advantage was highest at 75% SM and lowest at 25% SM. Furthermore, compared to the ET-based approach, it was demonstrated that the SM-based method conserved approximately 18.2% of irrigation water. As a result, when producing crops that require a lot of water, like tomatoes, in areas with limited water resources, SM-based irrigation scheduling is preferable to ET-based irrigation scheduling.

## 1 Introduction

### 1.1 Background of the study

The core of Ethiopia's economy is agriculture. Smart use of scientifically proven irrigation systems can reduce the crisis in food production regardless of climate change interventions. In particular, this practice is too important for the production of agricultural products in areas which have limited water supplies or where there are no irrigation facilities [1]. The sustainability of rural communities depends entirely on agriculture [2]. Restricted water system and inconsistent supply of water within a nation are essentially insufficient [3]. The primary issue stirring within the water system plans is unplanned water system application, especially over or beneath water system of crop areas. Relative irrigation supply (irrigation supply: crop water demand) at required level for 10 water system plans in Ethiopia ranges between 0.5 (deficient) to 5.0 (abundant) through January to May and between 0.8 (deficient) and 7.0 (overabundant) for the period between June to December [4]. Non-scheduled and the same sum of water application, notwithstanding of crop type [2] have been recorded in numerous cases. As of now, this subject is getting to be the major concern of researchers and researchers for the upgrade of crop productivity [5]. In arrange to extend agrarian efficiency, agriculturists, water system specialists, water assets directors and decision-makers recommended appropriate water system planning strategies.

The logical strategy of water system planning requires the timing, area and sum of water system, which can be conducted in two ways [6]. The first is specifically observing soil moisture with soil moisture sensors, and the other is utilizing weather data to depict the soil-water proportion through a soil-moisture balance approach. Some classify them into three “scientific” irrigation planning methods: plant, soil and climate-based [7,8]. Still, there is another classification that makes it a bit clearer than the others do. It separated the strategies into two fundamental groups: farmer's strategies and progressed strategies. Among these diverse classifications, soil based strategy and climate based strategy that are based on soil moisture and evapotranspiration are considered as conventional procedures [9]. During this research, these two strategies are compared to assess their efficacy on crop yield.

A common approach for evapotranspiration-based strategies is to observe ETc losses and augmentations due to irrigation and precipitation to balance the soil moisture accessible to plants [10]. This strategy consumes less water for the same yield and considered as a better approach irrigation compared with others [11,12]. Climatological parameter, especially, evapotranspiration is intensively used for high yield crop. When accurately applied, this planning practice has a long history of preserving water and maintaining crop yield and quality [13].

An SM-based schedule determines the soil water holding capacity between field capacity (FC) and permanent wilting point (PWP). Neutron scattering, tension meters, automatic irrigation controllers, electrical resistivity measures, capacitance sensors, watermark sensors, heat pulse sensors, and fiber-optic sensors are just a few of the many instruments available for irrigation scheduling [14,15]. All those sensors are broadly classified into two classes: volumetric water content type and soil water potential type. The percent volume of water in a unit volume of soil is measured by volumetric water content type, whereas, soil water potential is measured by soil water potential type [14]. Domain reflectometry (TDR), a type of time based dielectric sensors requires less field maintenance but has a greater potential for commercial adoption [15]. Volumetric water content and gravimeter were measured using TDR for calibration purpose.

Generally, the most popular methods are soil moisture-based [16] or evapotranspiration-based irrigation scheduling. But the demerit behind using those methods is the requirement of sufficient input data. Secondly, the results are never considered as scientific. Hence, the most favourable method is accounted for the method which provides suitability of their preference and on-site conditions [17]. Sound knowledge of technology, skill set, and unprejudiced information play a vital role in providing concrete solution to the problem. Conversely, comparison of the efficacy of these two methods of irrigation scheduling is based on their productivity as per the climatic condition [12,18,19]. For some crops, ET-based irrigation scheduling tools have been developed that delivered with equal potential yields with signiﬁcantly less water than some irrigation systems. This was proved by experiments on plots located in zone A (nomenclature zones A and B) that resulted in a 10.18 t/ha yield on checkbook (ET) and 9.699 t/ha on sensor-based treatment [20]. Under tis above circumstance, ET-based systems marginally give a better yield than sensor-based systems.

Prior research has not examined the productivity differences in yield between irrigation scheduling techniques through comparative studies. The degree of yield variance for the approaches is still absent from previous research studies. The outcomes differ between studies and locations for various characteristics and quantities of limiting factors (soil type, competency, crop, method, and climate) [21]. Despite the complexity of such studies, increase in constraining variables, appropriate planning and successful implementation of similar works against constraints is still vital. Augmenting agrarian planning, constructive management, and productive yield becomes a goal for the researchers to provide a better approach of irrigation facility under diverse climatic conditions.

Several researchers have been done irrigation scheduling by using either soil moisture (SM-based) [22] or evapotranspiration (ET-based) [23] in the study area and different places in advance with no preconditions to compare and choose. Regardless of the popularity of these two typical and representative methods in the region, studies were neither applied to compare the effectiveness of the methods nor combined them with the concept of deficit irrigation. In order to give customers options for managing their irrigated farms under current conditions, it is crucial to conduct a realistic performance evaluation of the approaches in terms of production under deficit irrigated crop.

In this study, Determinate Open pollinated, Roma VF tomato variety is selected. It is disease resisting crop as the short form ‘VF’ after the name ‘Roma’ indicates its ability to resist diseases called Verticillium wilt and Fusarium wilt. Further, management activities such as application of 150 kg/ha urea and 100 kg/ha of P_2_O_5_ (DAP) are suggested for the area. Proper weed control was taken place four times manually during each growth stages for proper management to attain the predicted yield of about 40ton/ha [24].

## 2 Materials and methods

### 2.1 Study area description

**Fig. 1:**
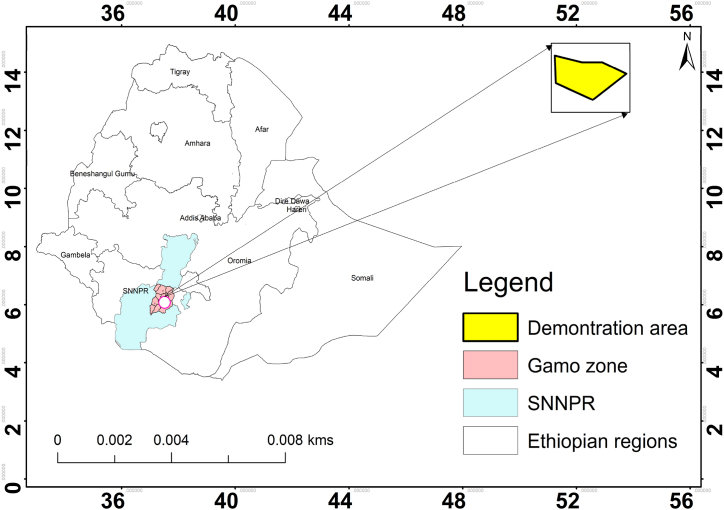
Location map of the study area.

**Fig. 2:**
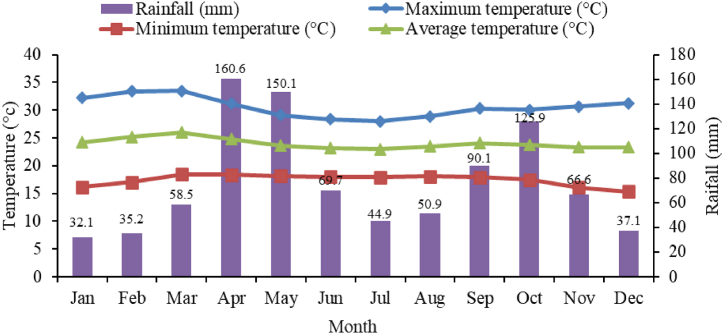
Climate data of the study area.

The research was piloted in Arba Minch, a city located in southern Ethiopia (Fig. 1), 500 km away from Addis Ababa, the capital city of Ethiopia. The research area is geographically located at 6°04′N latitude and 37° 34′ E longitudes and at an altitude of 1200 m amsl. The study area is bounded in the east and south-east by two Rift Valley Lakes, named Abaya and Chamo. The experiment was conducted from May to September 2022. Historical rainfall and temperature data for thirty years (1987–2018) (Fig. 2) were analyzed to comprehend the past climatic scenario especially, the dry climatic condition to conduct this research.

The only available meteorological station was located in Arba Minch University which provided the daily weather data like, temperature, rainfall, sunshine hours, wind speed and humidity. Those data are the evapotranspiration computation parameters for ET-based scheduling.

The study area enjoys a bimodal climatic condition with an average anuual rainfall of 921.8 mm.

### 2.2 Pre-experimental activities

**Fig. 3:**
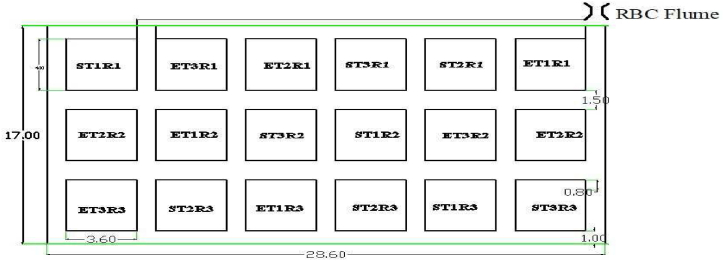
Layout of experimental field.

The land that was prepared for the experiment was 0.048 ha (28.6 m × 17 m). The total land was divided into 18 plots, each of 4 m × 3.6 m (14.4 m^2^) (Fig. 3). Each plot was distributed into 5 furrows with an 80 cm spacing, 20 cm deep, and 35 cm wide. The slope of the furrows is kept as 0.2% (vertical distance (m) to horizontal distance (m)). The crop was planted at the side of the ridge with 40 cm and 80 cm between plants and rows, respectively. Seeds were sown in nursery bed of 5 m long, 1 m wide, and 25 cm raised bed within 20 cm spaced line.

#### 2.2.1 Soil sample collection and analysis

**Fig. 4:**
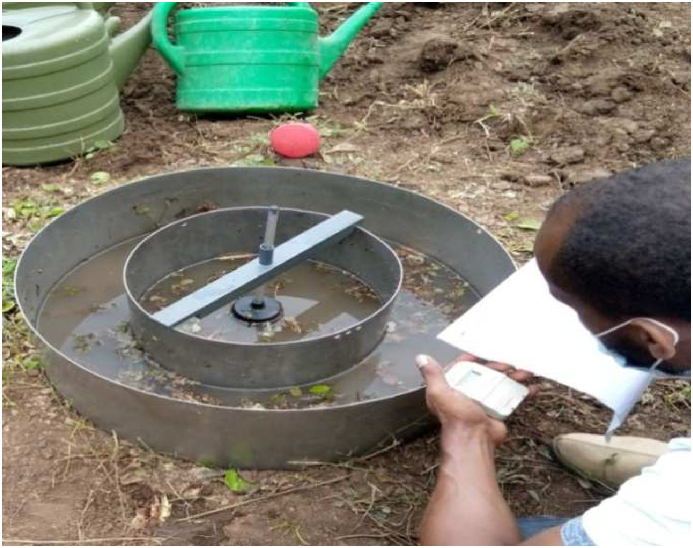
Soil infiltration test carried out in the field.

**Fig. 5:**
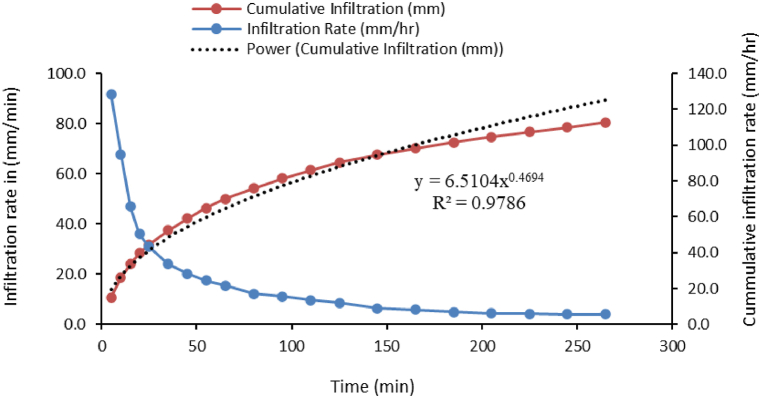
Infiltration test graph.

For the laboratory analysis, nine representative locations with soil bulk density and texture measurements from depths of 0–20, 20–40, and 40–60 cm were taken into account. The texture was measured using a Bouyoucos hydrometer, and the soil infiltration was measured using a double ring infiltrometer on a bare field with outer and inner diameters and heights of 53 cm, 32 cm, and 25 cm, respectively (Fig. 4, Fig. 5).

### 2.3 Treatments

Soil moisture for SMT1, SMT2, SMT3 was taken as 100%, 75%, and 50% respectively, whereas, ETT1, ETT2 and ETT3 represented for the same percentages respectively. The comparative treatments resulted from the both sides are SMT1 = ETT4 = 100%, SMT2 = ETT5 = 75%, and SMT3 = ETT6 = 50% requirement. Six treatments were irrigated by three levels of applications totaling eighteen on both the sides by Randomized Complete Block Design (RCBD) plots (Table 1).

**Table 1:** Treatments, application level, and plot, respectively.

SM Treatments	ET Treatments
SMT1, 100%, 1	ETT1, 100%, 1
SMT1, 100%, 2	ETT1, 100%, 2
SMT1, 100%, 3	ETT1, 100%, 3
SMT2, 75%, 1	ETT2, 75%, 1
SMT2, 75%, 2	ETT2, 75%, 2
SMT2, 75%, 3	ETT2, 75%, 3
SMT3, 50%, 1	ETT3, 50%, 1
SMT3, 50%, 2	ETT3, 50%, 2
SMT3, 50%, 3	ETT3, 50%, 3

Note: SM and ET are soil moisture and Evapotranspiration based plots; T1—T6 represents treatments, Numbers 1, 2 & 3 are 1st 2nd and 3rd plots; 100%, 75% and 50% are deficit levels.

## 3 Irrigation scheduling methods

### 3.1 ET-based irrigation scheduling

Daily Eto was calculated using FAO Penman-Monteith method from the available meteorological data of the area [19,21]. Melkasa research center provided the specific crop coefficient (Kc) for similar agroclimatic conditions [22]. Crop evapotranspiration is calculated using Equation (1) [23].

(1) ETc=KcxEto

ET-based irrigation scheduling is carried out for soil water balance estimation based on the water lost by evapotranspiration and its replacement through irrigation or effective rainfall. The balance was performed for row crops using Equation (2).

(2) CSWC=PSWC+Inet+Pe‐ETc

Where, CSWC = Current soil water content (today, mm), PSWC = Previous soil water content (yesterday, mm), Pe = Effective rainfall since yesterday (mm), Inet = Net irrigation since yesterday (mm), ETc = Crop evapotranspiration (mm/day)

The first stage in creating the water budget was figuring out how much water was in the soil (PSWC). PSWC is typically restored to field capacity one day before to the first irrigation. Equation (3) is used to determine the net irrigation water requirement [19].

(3) Inet=ETc‐Pe

Where, Inet = Net irrigation requirement (mm), Pe = Effective rainfall (mm), ETc = Crop evapotranspiration (mm).

Of the entire 117.6 mm of rainfall that were recorded during the trial period, the meteorological data shows that 58.9 mm of rainwater is efficiently connected to the crop root zone. The effective rainfall was calculated by comparing the rainfall and the crop water consumed (ETc), assuming that there was no input water other than rainfall. Pe equals rainfall if the crop's ETc for the day is more than the rainfall; else, Pe equals ETc. Equation (4) calculates the gross irrigation water need to make up for various losses.

(4) $$\text{GIR}=\frac{\text{Inet}\;}{\text{Ea}}$$

Where, GIR = Gross irrigation requirement (mm), Ea = Efficiency of application (%), and Inet = Net irrigation requirement of water to the field (mm).

### 3.2 SM-based scheduling

For SM-based irrigation, the amount of irrigation water used was determined by the root zone's moisture content at the moment of sampling. The various calculation to generate from the equations are presented in Equation (5) -7 [24].

The total available soil water was computed using Equation (5).

(5) TAW=(FC–PWR)xRD

Where, TAW = Total available soil water (mm), FC = Field capacity (%), PWP = Permanent wilting point (%), RD = Effective root depth of the crop (mm).

Readily available soil water was computed by Equation (6).

(6) RAW=MAD*TAW

Where, RAW = readily available water (mm), MAD = maximum allowable depletion (mm), TAW = total available soil water (mm).

Initiating the irrigation to bring soil moisture level from specific moisture deficit of soil to field capacity during each irrigation event was determined depending on daily depletion values is depicted in Equation (7) [22].

(7) $$\text{dn}=\frac{\left(\text{FC}‐\theta i\right)}{100}\text{RD}*\rho b$$

Where, dn = Net water applied (mm), FC = Field capacity (%), i = Actual soil moisture content using TDR (%), RD = Effective root depth (mm), ρb = Apparent specific gravity.

### 3.3 Discharge measurement (RBC flume)

**Fig. 6:**
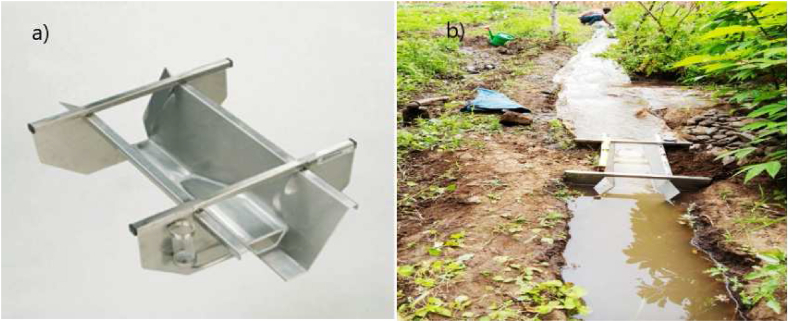
RBC flume (a), discharge measurement (b).

Irrigation water is allowed to pass through a submerged circular pipe and then to the ReplogleBos-Clemmens (RBC) flume (Fig. 6 (a)).

The water level (SH1) in the flume was measured in order to calculate the flow rate (Fig. 6(b)). Using Equation (8), the sill-referenced water level (SH1) was translated to flow rate. The table was chosen for discharge measurement out of the three options due to its ease of use.

(8) $$Q=7E‐07x{\left(\text{SH}1\right)}^{3}+0.000626\;x\;{\left(\text{SH}1\right)}^{2}+0.01569x\left(\text{SH}1\right)‐0.0665$$

Where, Q = Discharge (l/sec), SH1 (sill referenced water level) measured in mm.

**Fig. 7:**
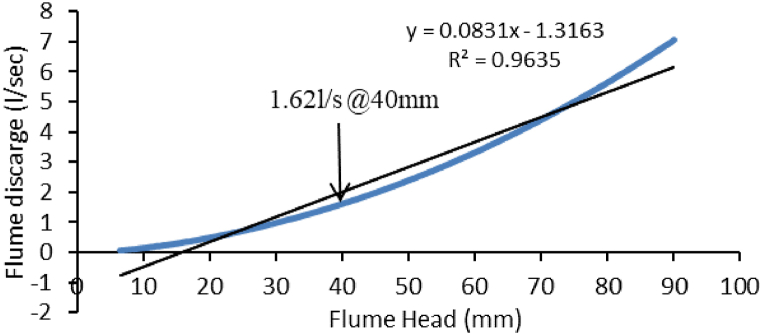
The relationship between RBC flume discharge (l/sec) and head (mm).

The flume was calibrated to a discharge of 1.62 l/s for SH1 of 40.0 mm (Fig. 7).

The time (min) of water application to each furrow is calculated using Equation (9) [25].

(9) $$T=\frac{\text{GIR}\;*\;L\;*\;W}{60Q}$$

Where, T = Application time (min), GIR = Gross depth of water applied (mm), L = Furrow length (m), W = Furrow spacing (m), Q = Flow rate (l/s)

### 3.4 Application efficiency (Ea)

A field experiment measuring the time of water recession (tr) in minutes, the distance of water advance (x) in meters, the time of water advance (tx) in minutes, the time of opportunity (To) in minutes, and the soil infiltration data was used to calculate the application efficiency. It is input for the determination of depth of infiltration (Equations (10), (11))).

(10) $$I={\text{aTo}}^{b}$$

Where, I = Cumulative depth of infiltration (mm), To = Time of opportunity (min), a & b = Constants obtained from the infiltration equation.

(11) To=tr‐tx

Where, tr = Time of recession (min), tx = Time of advance (min).

The depth of irrigation application water was determined using Equation (12).

(12) $$\text{Dapp}=\frac{1000*Q*\text{tco}}{A}$$

Where, Dapp = Depth of application (mm), Q = Discharge applied (m^3^/min), tco = Time of cut off (min), A = Furrow area (m^2^)

Considering the depth of irrigation application, application efficiency (Ea) was determined (Equation (13)).

(13) $$\text{Ea}=\frac{I\;}{\text{Dapp}}$$

Where, I = Average depth of infiltration stored in the root zone, Dapp = Depth of application.

### 3.5 Crop data collection and harvest

Five randomly chosen and tagged plants were used to gather yield and agronomic data at different phases of growth. Plant height (in centimeters), number of branches, number of fruits/plant, and number of flowers/plant were compared using agronomic metrics. In 2022, four harvests were conducted between September 8 and September 29. Fruit length (cm), fruit diameter (cm), and fruit weight (g) are examples of yield qualities. Marketable yield (ton/ha) and non-marketable yield (ton/ha) make up the total fruit production. The approaches' net advantages with respect to yield and applied water were also calculated.

### 3.6 Data analysis

After organizing and averaging the field data using Microsoft Excel, a significant difference between the measured parameters was examined using the growth parameters mentioned above and ANOVA (statistics trial 10) software. Using the least significant difference and the standard operating procedures [20] for randomized full block design, the mean square separation was carried out.

## 4 Result and discussion

Two irrigation-scheduling methods were comparatively evaluated for their agronomic characteristics, yield attributes, and yield. The effects of the methods on deficit irrigated crops and the net benefit of the methods regarding yield were determined.

The soil characteristics of the study area is predominantly clayey in nature as determined from Boyouncus method. The average range between FC and PWP available to the crop was 15.9%, which is within the tolerable limit [19].

The soil's average pH is 7.30, which is neutral and suggests that it is suitable for growing crops [26,27]. The site's electrical conductivity (EC), which has an average value of 2.14 dS/m, is the same. According to research, non-saline soils with an EC of less than 4 dS/m are good for crops [28].

5.7 mm/h is the fundamental infiltration rate at which the infiltration rate becomes almost constant. The rate of soil infiltration varies from 1 mm/h to 5 mm/h in certain cases [28]. **4.1 Irrigation Scheduling**.

The net irrigation requirement was determined based on Equations (3), (4)) (Table 2).

**Table 2:** Soil Properties of the area.

Depth cm	Sand %	Silt %	Clay %	Texture	Bulk density, g/cm^3^	FC, %	PWP, %	pH	EC, dS/m
0–20	28	31	41	Clay	1.11	38.71	20.13	7.29	2.46
20–40	30	27	43	Clay	1.13	34.30	19.50	7.31	2.03
40–60	22	34	44	Clay	1.15	31.52	17.20	7.30	1.93
Average	26.67	31.33	42	Clay	1.13	34.84	18.94	7.30	2.14

**Fig. 8:**
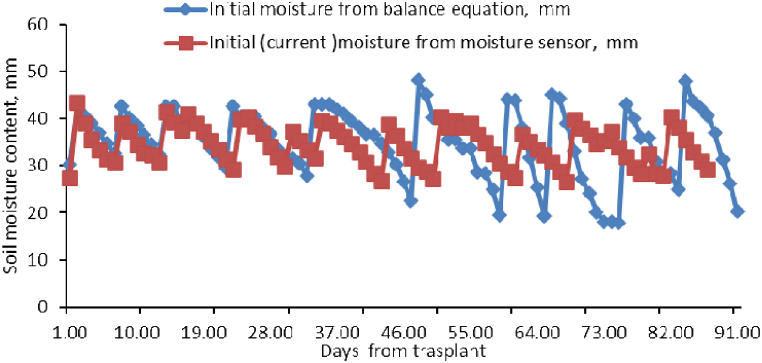
Soil moisture variations in scheduling methods.

While additional irrigation is required near the end of the season, the balance technique is not required when using moisture-based irrigation. These conditions would imply that, occasionally, the assumption-based balance could continue to decrease regardless of the soil's actual moisture level. Fig. 8 shows a comparison of soil moisture variance in SM-based and ET-based scheduling methods.

**Fig. 9:**
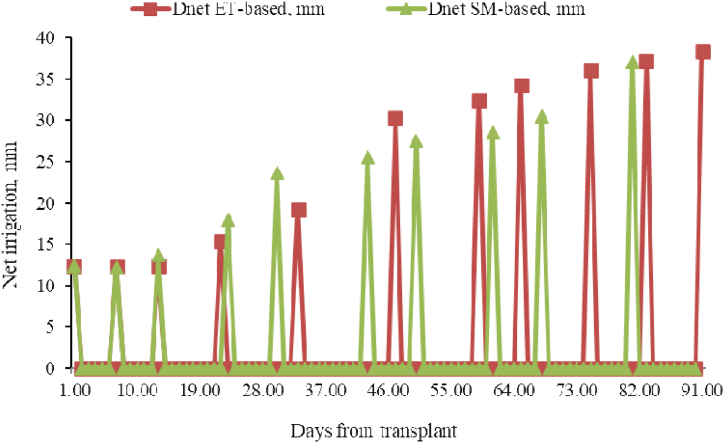
Net irrigation comparison for scheduling method.

The net irrigation water applied in SM and ET-based are 229.1 mm and 280 mm, respectively (Table 4, Fig. 9).

During the trial season, 117.6 mm of rainfall were reported across the whole ET-based timeframe. Of this, the root zone is able to effectively store 58.9 mm of rainfall. Using a moisture sensor, the effective rainfall on an SM-based system is calculated from the variation in soil moisture interpretation prior to and immediately following rainfall. 37.7 mm of water were applied to the root zone during this time.

Table 3s results show that, in comparison to the ET-based approach, the SM-based approach conserved 18.2% of the water. This suggests that the climate-based approach could not yield as good of findings as direct moisture monitoring. The outcome is consistent with the research by Ref. [28], which found that using a temporal domain transmissometry (TDT) sensor-based approach saved 16% more water than using ET-based irrigation recommendations. Furthermore [12], found that in tomato-growing fields, SM-based methods saved 31% more water than ET-based methods. Even if the results show that SM-based approaches have advantages, the amount of savings varies among all findings, including the current one. Numerous factors, including geographic and seasonal variations, crop and soil types, irrigation methods, the degree of experience of irrigation managers, application technology, and climatic conditions, may contribute to the discrepancies observed in the findings [21].

**Table 3:** Irrigation schedules.

No of Ir	SM-based			ET-based		
	Date of Ir	Inet (mm)	Ig(mm)	Date of Ir	Inet (mm)	Ig (mm)
Ir 1	21-Jun	12.4	19.0	21-Jun	12.4	19.0
Ir 2	27-Jun	12.1	18.7	27-Jun	12.3	18.9
Ir 3	03-Jul	13.7	21.2	03-Jul	12.4	19.0
Ir 4	13-Jul	18.0	27.6	12-Jul	15.4	23.7
Ir 5	20-Jul	23.7	36.4	23-Jul	19.2	29.5
Ir 6	02-Aug	25.6	39.3	06-Aug	30.3	46.6
Ir 7	09-Aug	27.6	42.4	18-Aug	32.4	49.8
Ir 8	20-Aug	28.5	43.9	24-Aug	34.2	52.6
Ir 9	27-Aug	30.5	47.0	03-Sep	36.1	55.5
Ir 10	09-Sep	37.1	57.0	11-Sep	37.2	57.2
Ir 11	–	–	–	19-Sep	38.4	59.1
	Sum	229.1	352	Sum	280	430.9

Note: Ir is irrigation, Inet is net irrigation, Ig gross irrigation, and Dates are on Gregorian calendar.

### 4.1 Effect of irrigation scheduling methods on agronomic characteristics and yield attributes

Yield performance is a result of agronomic characteristics and yield attributes. The effects of irrigation scheduling methods on agronomic characteristics are shown in Table 5. The results indicate that the characteristics are not significantly varying for the methods at (p ≤ 0.05). The observed variation in the table is only for water application levels.

**Table 4:** Net requirement of irrigation water.

Treatments	NIR(mm)	GIR(mm)
SMT1	229.1	352.5
SMT2	171.8	264.4
SMT3	114.6	176.2
ETT4	280.0	430.9
ETT5	208.5	323.2
ETT6	139.0	215.5

Note: T1, T2 and T3 are for treatment one, two and three, respectively. CU is consumptive use.

**Table 5:** ANOVA for effects of irrigation scheduling methods on the agronomy.

Treatments	Height (cm)	No. of primary branches	No. of flowers
SMT1	93.4^a^	9.2^a^	46.0^a^
SMT2	93.1^a^	8.9^a^	45.0^a^
SMT3	84.0^c^	7.3^b^	39.2^b^
ETT4	94.3^a^	8.9^a^	47.2^a^
ETT5	92.1^ab^	8.9^a^	45.6^a^
ETT6	84.8^bc^	7.5^b^	40.0^b^
CV	4.77	3.21	2.77
LSD	3.518	0.2216	0.991

Note: Similar spellings indicate the non-significant difference between means.

The highest (94.3 cm) and lowest (84.0 cm) plant heights were recorded from SMT3 and ETT4, respectively. The highest value at SMT1 (93.4 cm) does not significantly vary from ETT4 (94.3 cm). The mean value of tomato height is within a range of 39.34 cm and 96.67 cm. This result was in agreement with the findings of [30] who reported that the plant height of different tomato varieties is within a range of 40.20 cm and 107.00 cm. From Tables 4 and it can be observed that the characteristics shown here are neither increasing nor decreasing for methods. That makes it impossible to reach a genuine conclusion on their relation to scheduling methods. Nevertheless, the study by Ref. [30] has clearly shown the effect of irrigation scheduling methods on the agronomical characteristics of the plants. Variation in results could most probably be from the effect of experimental repetition and the level of technology. That was two seasonal experiments aided by controllers for ET-based and automatic sensors for SM-based scheduling. Such differences could have a significant impact on the accuracy of results [31]. The characteristics show a decreasing trend toward the deficit level. Other variables evaluated for irrigation scheduling methods are yield attributes like number of fruits per plant, fruit weight (g), fruit length and diameter (cm) as presented in Table 6. If agronomic characteristics were varied for methods, yield attributes could vary. Accordingly, the results in the table indicate non-significant variation in attributes for scheduling methods.

**Table 6:** ANOVA for effects of irrigation scheduling methods on yield attributes.

Treatments	No. of fruit/plant	Fruit weight(g)	Fruit diameter(mm)	Fruit length (mm)
SMT1	48.9^a^	67.7^a^	42.7^a^	55.6^a^
SMT2	48.6^a^	66.7^a^	41.9^ab^	55.3^a^
SMT3	41.2^b^	45.9^b^	34.8^c^	45.1^b^
ETT4	48.8^a^	68.1^a^	41.2^ab^	56.4^a^
ETT5	47.6^a^	64.8^a^	40.8^b^	55.9^a^
ETT6	41.7^b^	43.5^b^	35.0^c^	46.5^b^
CV	4.03	4.45	2.47	4.26
LSD	1.518	2.155	0.793	1.813

Note: Similar spellings indicate a non-significant difference between means.

From the table, it can be observed that the attributes are decreasing toward deficit level. The average maximum fruit weight in SM and ET-based at SMT1 and ETT4 are 67.7 and 68.1g respectively. The weight is reduced with deficit levels to 45.9g and 43.5g in SMT3 and ETT6, respectively. The values in the table are in agreement with [32] who found that direct relation between the parameters and application levels.

### 4.2 Effect of irrigation scheduling methods on yield performance

Marketable, non-marketable and total yield were determined based on fruit lengths, diameter, weight and fruit health status. From the analysis, as indicated in Table 7, these variables have not shown significant differences for irrigation scheduling methods at P < 0.05. Consequently, this has resulted in non-significant variations in yield for the methods.

**Table 7:** ANOVA for the effect of irrigation scheduling methods on yield performance.

Treatment	Marketable yield (ton/ha)	Non-marketable yield (ton/ha)	Total yield (ton/ha)
SMT1	41.0^a^	1.70^c^	42.70^a^
SMT2	38.7^ab^	3.17^bc^	41.87^a^
SMT3	25.2^c^	4.20^ab^	29.40^b^
ETT4	40.8^a^	1.80^c^	42.50^a^
ETT5	38.2^b^	3.40^bc^	41.63^a^
ETT6	24.3^c^	4.60^a^	28.90^b^
CV	3.92	16.8	2.71
LSD	1.142	0.472	0.943

Note: Similar spellings indicate the non-significant difference between means.

The highest marketable yield observed at SMT1 (41 t/ha) and ETT4 (40.8 t/ha) as shown in Table 6 are similar at (P < 0.05). Similarity of yield for different scheduling methods may indicate that there could be another external factor that could affect yield performance. Anyway, cross-comparison of marketable yield at SMT1, SMT2 and SMT3 are 0.7%, 1.2% and 2.1% higher than the corresponding ETT4, ETT5 and ETT6, respectively. This shows a little advantage of SM-based over ET-based for marketable yield. The result agrees with a finding by Ref. [12] that conducted a comparative evaluation of these two scheduling methods for total marketable yields and reported no significant difference. Regarding the level of irrigation, marketable yield decreased with reducing the level. This was justified by other findings [30].

Non-marketable yield includes small-sized, malformed, diseased and injured fruit. The values in SM-based at SMT1, SMT2 and SMT3 are 5.5%, 5.8% and 8.7% lower than the corresponding ET-based treatments at ETT4, ETT5 and ETT6, respectively. For both methods, more than half of the total non-marketable yield is observed under 50% application level. This indicates highly water-stressed crops are vulnerable to non-marketability. For the level of application, decreasing irrigation level from 100% to 75% increased non-marketability to some extent. However, decreasing the level from 100% to 50% increased it significantly. As the crop faces a high water deficit, the process can be affected negatively through reduced evapotranspiration resulting in loss of quality, size and weight [29].

The overall yield, which is the product of marketable and non-marketable yield, yield characteristics, and agronomy, is not significantly different for scheduling approaches at P < 0.05 (Table 6). It declined in the direction of a greater deficit, as further research revealed [30]. The average total yield of 39.2 t/ha from the SM-based experimental field is comparable to the ET-based yield of 39 t/ha. The total output could not change because factors including soil, climate, and agronomic traits are identical [30]. Comparably, two zonal tests in zones A and B were conducted using the checkbook and sensor-based treatments [20]. According to the outcome in zone A, ET-based outperforms sensor-based somewhat, but vice versa at zone B. The combined average yield from the two approaches is almost identical to the estimated average yield (about 40 tons/ha) for the chosen open-pollinated Roma VF variety [31]. It is evident from the closely related results above that before making pretentious recommendations, these kinds of comparative studies must be conducted beforehand in the area and in other areas as well to see the real effects of irrigation scheduling methods on yield, yield attributes, and agronomic characteristics.

#### 4.2.1 Net benefit

The estimated saved water and yield reduction in percent for treatments are compared in Table 8. Results indicate that 50% water application, reduced yield by 38.6% and 40.4% in SM and ET-based treatments, respectively.

**Table 8:** Water saved against yield reduction.

Treatments	NIR(m^3^/ha)	Saved water(m^3^/ha)	Marketable(t/ha)	Yield reduction (%)
SMT1	2291.1	0.0	41.0	0.0
SMT2	1718.4	572.8	38.7	5.6
SMT3	1145.6	1145.6	25.2	38.6
ETT4	2779.6	0.0	40.7	0.0
ETT5	2084.7	694.9	38.2	6.3
ETT6	1389.8	1389.8	24.3	40.4

Reduction of irrigation water to 75% in SM and ET-based methods reduced only 5.6% and 6.3% yield, respectively compared to the control. It can be considered as tolerable yield reduction compared to yield loss recorded in 50% application. This is a direct indication of the dependence of yield on water application level. 50% application treatments could result in a 9%–46% yield reduction for different tomato cultivars [31]. This more or less agrees with the 38.2% and 40.4% yield reductions in Table 7. The net benefit obtained from treatments indicated the overall advantage of scheduling methods and appropriate treatment levels. Table 9 presents extra yield, land and final net benefit on yield from the saved water. The treatments SMT1 and ETT4 used full water so they have neither extra land nor extra yield.

**Table 9:** Net benefit comparison on yield.

Treatments	Extra land (ha)	Extra yield (t/ha)	Total yield (t/ha)	Net yield benefit (t/ha)
SMT1	0.00	0.0	41.0	0
SMT2	0.33	12.9	51.6	10.6
SMT3	1.00	25.2	50.2	9.2
ETT4	0.00	0.0	40.7	0
ETT5	0.34	12.7	50.9	10.2
ETT6	1.00	24.3	48.6	8.1

The results indicate SM-based method has slightly higher benefits than the corresponding ET-based treatments. For both methods, the maximum net benefit is from 75% application. This is because the treatment used 25% less water but it had a comparable yield compared to the control treatment. The maximum net benefit recorded for both SM (10.6 t/ha) and ET-based (10.2 t/ha) is from this treatment. Based on the results from Table 7, Table 8, the SM-based scheduling method with 75% application level can be an appropriate combination for higher net benefit on yield. SM treatments are competing ET treatments for yield (Table 3, Table 8) but save water. Considering the net benefit and improvement of agricultural water management in the area, the SMT2 could be more appropriate and profitable combination.

In the study area, these two irrigation scheduling practices are commonly applicable with inappropriate scheduling methods. Some of the farmers in the area were using the methods traditionally as a fixed calendar (ET-based) for example, 7 days irrigation interval for tomatoes and others are experiencing hand-feel method (SM-based) to or not to initiate irrigation. However, connecting farmers in the region with such modern soil moisture sensors and assisting their assumption-based experience by direct measuring, is necessary. The farmers need to get consultancy, awareness and training on interpretation of the technology by irrigation experts, researchers, agricultural extension and management class.

## 5 Conclusions

The experiment's net water application under the SM and ET-based irrigation scheduling approaches was 229.1 mm and 280.0 mm, respectively, according to the data. The study found that there was infrequently a significant difference (P < 0.05) between the irrigation scheduling methods utilized in the area during the examination period and the agronomic qualities, yield attributes, and crop yield. It was found that scheduling techniques had no discernible effect on the yield attributes. Because of this, coming to a true conclusion about how they relate to the methodologies is astounding. The overall average yield that was collected using ET-based approaches was 39.2 t/ha, while the yield obtained using SM methods was 39 t/ha, nearly equal. On the other hand, SMT2 showed the highest net benefit in comparison to yield, while ETT6 showed the lowest. Reduced deficit irrigation to 75% only 5.6% and 6.3% yield in the ET-based treatment and SM treatment, respectively, which is acceptable. Providing sensible use of available water during the irrigation seasons is the primary goal of implementing irrigation scheduling using suitable techniques. For tomato growers in water-scarce places, the SM-based irrigation scheduling approach with a 75% level of application (SMT2) may therefore be more suitable and lucrative. Therefore, in order for the producers to use these technologies to modernize their routine irrigation practices and save water while increasing production, it is imperative that they are connected to them.

## Data availability statement

The data that support the findings of this study are available on request from the corresponding author.

## CRediT authorship contribution statement

**Solomon Mathewos Boltana:** Formal analysis, Data curation, Conceptualization. **Tigistu Yisihak Ukumo:** Methodology, Investigation, Formal analysis, Conceptualization. **Tarun Kumar Lohani:** Writing – original draft, Visualization, Validation, Supervision, Resources, Project administration, Writing – review & editing. **Nahom Bekele Mena:** Investigation, Conceptualization. **Muluneh Legesse Edamo:** Supervision, Software, Resources, Methodology. **Matusal Arja Alaro:** Visualization, Validation, Project administration, Investigation. **Bereket Dora Doliso:** Visualization, Software, Methodology, Investigation, Formal analysis.

## Declaration of competing interest

The authors declare that they have no known competing financial interests or personal relationships that could have appeared to influence the work reported in this paper.
